# Analysis of Fish-Consumption Benefits and Safety Knowledge in a Population-Based Sample of Polish Adolescents

**DOI:** 10.3390/nu15234902

**Published:** 2023-11-23

**Authors:** Zofia Utri-Khodadady, Dominika Głąbska

**Affiliations:** Department of Dietetics, Institute of Human Nutrition Sciences, Warsaw University of Life Sciences (WULS-SGGW), 159C Nowoursynowska Street, 02-776 Warsaw, Poland; zofia_utri@sggw.edu.pl

**Keywords:** fish, fish consumption, nutrition, nutritional knowledge, benefits, risks, safety, food safety, adolescents, teenagers, children, youth

## Abstract

Inadequate fish consumption is common and may result from multiple reasons, especially in adolescents who are a population at particular risk of the negative consequences of not consuming the recommended amounts of fish. The aim of the study was to analyze the knowledge about fish-consumption benefits and safety in a population-based sample of Polish adolescents. The stratified random sampling was conducted within two stages: sampling of counties from all voivodeships in Poland (being the basic administrative units of Poland) and inviting secondary schools from the drawn counties to obtain a sample representative of all regions of Poland. The Computer-Assisted Web Interview (CAWI) method was applied to gather the data within the study, and a questionnaire concerning knowledge about fish-consumption benefits and safety with 20 true–false statements was applied. Among 1289 participants, the statement with the highest share of correct answers concerned fish being a source of protein (78.9%) and fish-derived fats being health promoting (77.0%). The statement receiving the least correct answers concerned the type of fatty acids found in fish (7.6%) and the risk of polychlorinated biphenyls (PCBs) in fish (20.5%). Participants who were female, older than 18, underweight, living in an urban environment, from a region far away from the sea and from comprehensive schools provided a higher share of correct answers than other subgroups (*p* < 0.05). Knowledge concerning fish-consumption benefits and safety among Polish adolescents is in many cases inadequate; thus, nutritional education is needed, especially among younger adolescents, those attending vocational schools, males and those living in a rural environment.

## 1. Introduction

Fish is an important food group in the human diet, being one of the best sources of long-chain omega-3 polyunsaturated fatty acids (LC-PUFAs), such as the eicosapentaenoic acid (EPA) and docosahexaenoic acid (DHA) which are rarely provided from other food products [1]. The EPA and DHA fatty acids are considered essential in the human diet as they cannot be produced by the human body to meet the physiological requirements [2]. What is more, fish play an important role in providing nutrients essential for the endocrine system such as iodine, selenium and vitamin D [3]. Meta-analyses show that a higher fish intake decreases the risk of myocardial infarction [4], stroke [5], metabolic syndrome [6], dementia [7], and depression [8], as well as that it reduces all-cause mortality [9]. Moreover, a recent study indicated pescatarians to have the lowest DNA damage compared to omnivores and vegetarians, suggesting that it might be some compounds found in fish that protect DNA molecules from damage [10]. Taking this into account, predictive model analyses suggest that the implementation of food strategies based on fish could play a vital role in providing global food and nutrition security [11].

Due to their beneficial nutritional value, fish are indicated in many national and international dietary recommendations. During the 2nd International Conference on Nutrition organized by the Food and Agriculture Organization of the United Nations (FAO) in 2014, fish was recognized as having a special role in nutrition and health [12]. The World Health Organization (WHO) [13], as well as the European Society of Cardiology (ESC) recommend to consume fish at least one to two times per week [14], while the American Health Association (AHA) recommends consuming fish at least two times per week [15]. In Europe, fish intake recommendations range from 100 to 482 g weekly and usually correspond to one to two portions of fish in a week [16], similar to the Polish recommendation of at least two portions of fish weekly [17]. Some countries, such as Spain, recommend consuming even more fish—at least two to four portions per week [18]. Also, the Mediterranean diet, which is confirmed to be protective against the major chronic degenerative diseases [19], consists of at least two portions of fish or seafood per week [20]. Therefore, food-based dietary guidelines emphasize the role of fish as an important element of a healthy diet, not to be replaced with other protein sources [21].

Despite the described recommendations and numerous health benefits of consuming fish, a decreasing trend in its consumption has been observed in the European Union (EU) since 2018 [22]. The average apparent consumption in all 27 EU countries in 2020 amounted to 23.28 kg per capita yearly, which corresponds to 448 g per capita weekly. However, fish intake varies greatly from country to country, while in Portugal in 2020 it was 57.67 kg/capita/year corresponding to 1.11 kg/week, in Poland it was more than four times less, at 13.33 kg/capita/year, which corresponds to 256 g/week [22]. The fact that fish intake varies greatly depending on the country is associated with a very diverse consumption of omega-3 fatty acids of seafood origin, including fish, among countries. Studies show that only 18.9% of the global population achieves the recommended intake of omega-3 from fish of at least 250 mg per day [23].

Not only does fish consumption differ between countries, but also individual differences are observed. The 2005–2010 National Health and Nutrition Examination Surveys (NHANES) revealed that the lowest fish consumption was observed among younger individuals, as well as individuals of lower income and education level [24]. It corresponds with the results of Polish studies indicating that in a group of adolescents 14.1% of them declared consuming fish less than once per week, and 26.2% of them declared that they did not consume fish at all [25]. In a group of female adolescents, 49.1% of them reported consuming fish not more than once per month [26]. Moreover, adolescents are indicated within especially vulnerable populations at particular risk of the negative consequences of inadequate fish consumption [27]. Just like for other food choices, the reasons for not adhering to the nutritional recommendations concerning fish consumption are numerous: its high price, lack of knowledge on preparation techniques, no national cultural traditions of consuming fish [28], as well as not favoring them as a food product [29]. The lack of preference for fish is indicated to result from fish bones and fish smell, but also from not being accustomed to consuming fish due to other food habits developed during childhood [30]. What should be noted is that consuming fish is sometimes perceived by some consumers as unsafe and posing a risk to the human health [31,32]. At the same time, some studies indicate that this belief is more prominent among young respondents compared with older age groups [31], which may limit the fish consumption in this population group.

In order to increase fish intake national educational campaigns concerning the benefits of fish intake were introduced in Poland in the years 2008–2009 by the Ministry of Agriculture and Rural Development, and were entitled ‘Fish affects everything’. According to the governmental data, an increase in fish intake was observed afterwards; the annual mean of fish intake in the year 2007 was 12.91 kg/capita/year, while in the year 2008 it was 13.67 kg/capita/year, and in 2009 it was 13.94 kg/capita/year [33]. However, not much is known about what Poles, including adolescents, know about specific fish-intake benefits. What is known is that, according to a European repeated consumer survey compared to 2004, in 2008 adult Poles showed a more-positive attitude towards fish, as well as increased knowledge regarding fish [34]. It was suggested that this could have resulted from national policy efforts, confirming that educational campaigns can also influence people’s attitude towards fish. Importantly, the belief that consuming fish is healthy seems to be correlated with the frequency of eating fish [35].

Taking into account the serious problem of inadequate fish consumption, in their recommendations the joint FAO and WHO consultation indicated that it is necessary to emphasize the benefits of fish consumption, as well as to develop and evaluate communication strategies that both minimize risks and maximize benefits resulting from fish consumption [13]. Based on the described current state of knowledge and the lack of comprehensive studies assessing the problem in the Polish population, the aim of the study was to analyze the knowledge about fish-consumption benefits and safety in a population-based sample of Polish adolescents.

## 2. Materials and Methods

### 2.1. Ethical Statement

The study was carried out at the Department of Dietetics, Warsaw University of Life Sciences (WULS-SGGW). The study was conducted according to the guidelines laid down in the Declaration of Helsinki, while participants and their parents/legal guardians provided their informed consent for participation in the study. All procedures involving human subjects received the approval of the Ethics Committee of the Central Clinical Hospital of the Ministry of Interior and Administration in Warsaw (2/2021; approval date: 20 January 2021), and the study was conducted from May to July 2021. 

### 2.2. Studied Group

The study was conducted using a population-based sample of Polish secondary school students, which was gathered within a stratified sampling of secondary school students from counties based on the online National Register of schools and educational establishments of the Polish Ministry of Education and Science [36]. The following types of secondary schools were taken into account: comprehensive high schools, specialized high schools, vocational schools, technical schools and visual arts high schools. The net enrollment rate for those five types of secondary schools is 90.83% [37], so the sampling within schools was stated to allow the opportunity to gather a national population-based sample.

In order to obtain a national sample of adolescents in this age group which would be as representative as possible, stratified random sampling was conducted within two stages: (1) random sampling of counties from all voivodeships in Poland (being basic administrative units of Poland), and (2) inviting secondary schools from all counties sampled within the previous stage. Within the applied recruitment procedure (1) from each voivodeship (16 voivodeships in Poland), 30% of counties were sampled (115 counties sampled in total), and (2) from each county, all secondary schools were sampled (1357 secondary schools sampled in total).

Headteachers from each selected school received an email invitation for the school to take part in the study, as well as information about the aim and the scope of the study. Finally, 32 secondary schools participated, as headteachers expressed their willingness for the school to participate and gathered informed consent of students and their parents/legal guardians. Participation in the study was voluntary. Students willing to take part were sent an electronic link to the questionnaire prepared in Google Forms, while using the Computer-Assisted Web Interview (CAWI) method to gather the data within the study. They were also sent guidelines on how to carry it out, e.g., that the parents/legal guardians, as well as teachers should not help the students fill in the questionnaire, or that it is possible to fill it in on a mobile phone, but it is more comfortable to do it with the use of a computer. The dedicated questionnaire was anonymous and did not collect any data that would allow the identification of the respondents; however, it allowed for the verification of the inclusion/exclusion criteria.

The inclusion criteria were as follows:

- Adolescents aged 14–22 years;
- Attending one of the five given types of secondary school in Poland: comprehensive high school, specialized high school, vocational, technical or visual arts high school;
- Attending a secondary school sampled within the study;
- Informed consent to participate (verified by the headteacher);
- Informed consent of parent/legal guardian for participation (verified by the headteacher).

The exclusion criteria were as follows:

- Any missing data within the questionnaire once completed;
- Any unreliable answers within the questionnaire once completed.

**Figure 1:**
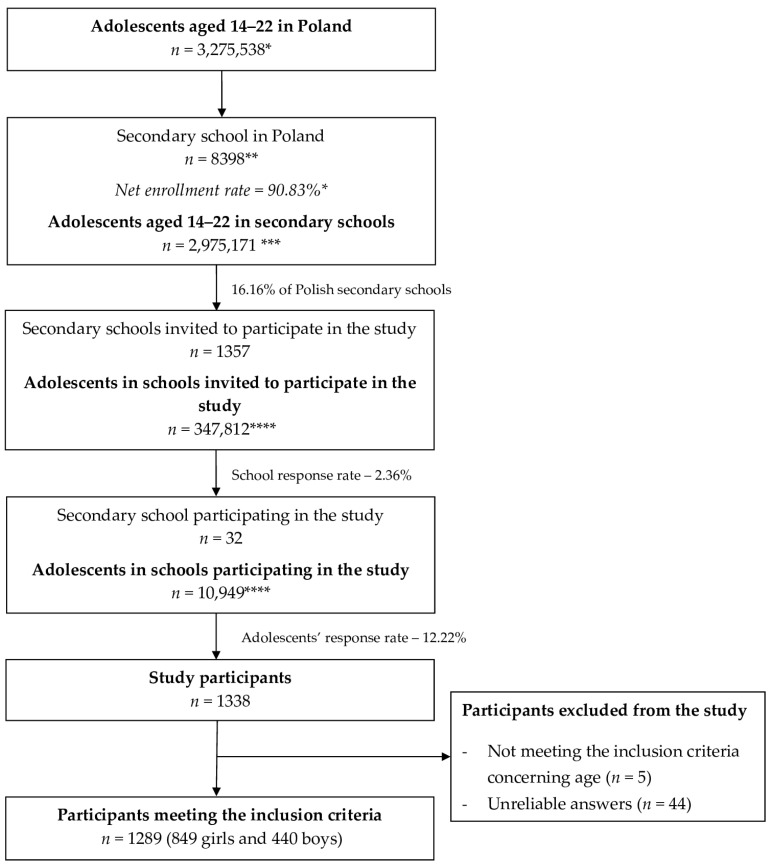
Detailed sampling procedure and recruitment of the studied group. * based on Statistics Poland data for the year 2021 [39]; ** based on the National Register of schools and educational establishments of the Polish Ministry of Education and Science for the year 2021 [36]; *** calculated based on Statistics Poland data for the year 2021 [39]; and **** calculated based on the National Register of schools and educational establishments for the year 2021 of the Ministry of Education and Science [36].

The respondents comprised pupils from all seven Polish macroregions (NUTS 1 units in the statistical division of Poland from the year 2021 [38]). The total sample of secondary school students gathered within the study was 1289, and the sampling procedure is presented in Figure 1.

### 2.3. Applied Questionnaire and Data Collection

The questionnaire concerned knowledge about fish and included 20 statements—both correct and incorrect ones to be assessed as true/false. The questionnaire comprised statements from a study by Burger and Gochfeld [40], examining knowledge on fish-consumption benefits and safety risks, as well as additional statements included after transcultural adaptation of the questionnaire. Due to the fact that the questionnaire by Burger and Gochfeld [40] was previously not applied to the Polish population and was previously not translated into Polish, the Polish version was to be developed. The questions were translated from English, according to the recommendations of the WHO [41], while transcultural adaptation was applied, if necessary. The process was applied in three stages: (1) forward translation into Polish (by a native Polish-speaking and English-fluent researcher familiar with the discipline and aim of the study); (2) backward translation into English (by other native Polish-speaking and English-fluent researcher, but who was not familiar with the aim of the study); and (3) expert-panel polishing of the questionnaire to keep the equivalence of the original questionnaire in the conceptual, semantic, idiomatic and cultural area (by a panel of native-Polish-speaking and English-fluent researchers familiar with the discipline and aim of the study).

The final questionnaire, in its Polish version developed within the study, includes 12 true statements and 8 false statements, as presented in Table 1. It covers the following issues: the content of nutrients in fish (statements 1–5, 9, 11, 19), their influence on human health (statements 6–8, 10), the health risks associated with consuming fish (statements 12, 13, 16, 18, 20), and the nutritional recommendations concerning fish (statements 14, 15, 17). They were intentionally placed in a disorganized order, with not all statements from one field in a row, to reduce the question order bias. Similarly, intentionally, the statements were formulated in a way to include a similar amount of correct and false statements to reduce the confirmation bias. Taking this into account, nine true statements from the questionnaire by Burger and Gochfeld [40] (statements 1, 5, 6, 8, 12, 13, 16, 18, 20) were taken without any changes, two true statements from the questionnaire by Burger and Gochfeld [40] were reversed and adopted to be used as false statements (statement 4, 7), and one was specified (statement 3). One complex statement with several parts was divided into two separate ones—a true statement (10) and a false statement (statement 11). Additional statements were developed in order to access knowledge on issues other than those in the questionnaire by Burger and Gochfeld [40]—one of them was formulated as true (statement 15), and the other five were formulated as false (statements 2, 9, 14, 17, 19).

Participants were asked whether in their opinion the given statements are true or false. They had the possibility to choose the ‘I don’t know’ answer as well.

Furthermore, questions concerning gender (close-ended question), exact age (open-ended question), height (open-ended question), weight (open-ended question), secondary school (open-ended question), place of residence (open-ended question) and fish consumption were included in the questionnaire.

Prior to the main research, a pilot study among 28 students from two secondary schools was conducted in order to ensure that all questions are comprehensible and that there are no technical problems. These students were from schools which were not sampled for the main research. The pilot study confirmed that the developed questionnaire is understandable and no other problems existed, so the questionnaire was not changed after the pilot study.

### 2.4. Statistical Analysis

For data analysis, the collected answers were grouped into ‘correct’ and ‘incorrect’ which comprised the ‘incorrect’ as well as the ‘I don’t know’ answer.

The normality of the distribution of the obtained data was verified using the Shapiro–Wilk test, and the groups were compared using the chi2 test (comparison of the share of respondents in sub-groups). The accepted level of significance was *p* ≤ 0.05. The statistical analysis was conducted using Statgraphics Plus for Windows 5.1 (Statgraphics Technologies Inc., The Plains, VA, USA).

For sub-groups analysis, all participants were divided into the following sub-groups depending on the following:

- Gender: Female and male.
- Age: Minors (less than 18 years of age) and adults (18 years of age or more).
- Body mass: Underweight, proper body mass and excessive body mass; it was defined based on the Body Mass Index (BMI), while for adults the standard cut-offs by the WHO were applied (18.5–25 kg/m^2^ as proper body mass) [42]. For minors, the Polish growth-reference cut-offs were applied [43] (5th–85th percentile as proper body mass) [44].
- Place of residence: Rural environment (village as a place of residence) and urban environment (city as place of residence).
- Location of the region of residence in relation to the Baltic Sea (being the only sea in Poland, as fish availability is commonly related to the seaside proximity [45]): regions situated by the sea (north and north-west macroregions of Poland) and away from the sea (central, Masovian, south-west, south and east macroregions of Poland)—it was defined based on the macroregion categories assumed by the Central Statistical Office in Poland [46].
- Type of school: Comprehensive school (comprehensive high schools and specialized high schools) and vocational school (vocational schools, technical schools and visual arts high schools).

## 3. Results

The characteristics of the studied group are presented in Table 2. Proper body mass was observed in 69.7% of all participants of the study, in 70.0% of the females and 69.3% of the males. Being underweight was seen in 7.0% of all participants, in 8.8% of the females and in 3.4% of the males, while excessive body mass was present in 23.3% of all participants, in 21.2% of the females and 27.3% of the males (assessed based on BMI with standard cut-offs by the WHO for adults [42] and Polish growth-reference cut-offs for minors [43]).

The number of correct and incorrect answers provided by the population-based sample of Polish secondary school students within the studied issues of fish-consumption benefits and safety concerns is presented in Table 3. The statement with the highest share of correct answers was the one concerning fish being a source of protein (78.9%), followed by fish-derived fats being health promoting (77.0%), and the recommendation for fish to be consumed by children and adolescents (74.9%). The statement obtaining the least correct answers was the reverse and ‘dummy’ statement concerning the type of fatty acids found in fish (7.6%), followed by the statement about the risk of polychlorinated biphenyls (PCBs) in fish (20.5%), and another reverse and ‘dummy’ statement regarding the fact that some fish have more fat than others (22.3%).

The number of correct and incorrect answers provided by the population-based sample of Polish secondary school students within the studied issues of fish-consumption benefits and safety concerns in sub-groups stratified by gender is presented in Table 4. Compared to males, female adolescents provided a higher share of correct answers to the statements about fish being a source of protein (81.3% vs. 74.3%; *p* = 0.0047), fish being good for health (73.9% vs. 65.9%; *p* = 0.0035), for the heart (66.3% vs. 60.5%; *p* = 0.0433), as well as about the recommendations for adolescents (79.3% vs. 66.6%; *p* < 0.0001) and for pregnant women to consume fish (39.5% vs. 29.1%; *p* = 0.0003). Male participants provided a higher share of correct answers to the statement concerning the risk of polychlorinated biphenyls (PCBs) in fish (24.8% vs. 18.3%; *p* = 0.0075).

The number of correct and incorrect answers provided by the population-based sample of Polish secondary school students within the studied issues of fish-consumption benefits and safety concerns in sub-groups stratified by age is presented in Table 5. Adult participants provided a higher share of correct answers compared to minor participants concerning the statements about fish being a source of protein (84.5% vs. 77.2%; *p* = 0.0088), some fish having more fat than others (26.6% vs. 21.0%; *p* = 0.0492), fish consumption being good for health (78.8% vs. 68.9%; *p* = 0.0012), for the heart (70.7% vs. 62.4%; *p* = 0.0107), and for the brain (70.7% vs. 64.0%; *p* = 0.0394), as well as the recommendation to consume fish at least twice a week (40.1% vs. 31.4%; *p* = 0.0064).

Table 6 presents the number of correct and incorrect answers provided by the population-based sample of Polish secondary school students within the studied issues of fish-consumption benefits and safety concerns in sub-groups stratified by the body mass of the studied adolescents. Compared to the other two sub-groups, underweight participants provided a higher share of correct answers to the statements about some fish having more fat than others (27.8% vs. 23.4%—proper body mass and 17.3%—excessive body mass; *p* = 0.0404) and fish not being a source of vitamin C (35.6% vs. 31.8%—proper body mass and 21.7%—excessive body mass; *p* = 0.0018).

The number of correct and incorrect answers provided by the population-based sample of Polish secondary school students within the studied issues of fish-consumption benefits and safety concerns in sub-groups stratified by rural/urban environment is presented in Table 7. Adolescents living in an urban environment provided a higher share of correct answers to the statements about some fish having more fat than others (25.0% vs. 19.8%; *p* = 0.0273), fish consumption being good for the brain (70.0% vs. 61.5%; *p* = 0.0016), fish-derived fatty acids being good for health (70.2% vs. 63.0%; *p* = 0.0072), and about the risk of contaminants in fish (64.8% vs. 56.0%; *p* = 0.0016).

Table 8 presents the number of correct and incorrect answers provided by the population-based sample of Polish secondary school students within the studied issues of fish-consumption benefits and safety concerns in sub-groups stratified by the location of the region of residence in relation to the Baltic Sea. Participants living in regions situated far away from the sea provided a higher share of correct answers to the statements about fish not being a source of fiber (33.3% vs. 22.1%; *p* = 0.0001), some fish having more fat than others (24.9% vs. 16.1%; *p* = 0.0008), fish not being a source of vitamin C (32.9% vs. 22.1%; *p* = 0.0001), the recommendation for children and adolescents to consume fish (77.6% vs. 68.8%; *p* = 0.0011), and the risk of mercury in fish (35.4% vs. 24.0%; *p* = 0.0001).

The number of correct and incorrect answers provided by the population-based sample of Polish secondary school students within the studied issues of fish-consumption benefits and safety concerns in sub-groups stratified by the type of school the participants of study attended is presented in Table 9. Adolescents attending comprehensive school provided a higher share of correct answers to the statements about fish not being a source of fiber (37.9% vs. 25.7%; *p* < 0.0001), some fish having more fat than others (33.9% vs. 16.1%; *p* < 0.0001), fish not being a source of vitamin C (38.1% vs. 25.2%; *p* < 0.0001), fish consumption being good for the heart (68.4% vs. 62.1%; *p* = 0.0305) and the brain (71.5% vs. 62.4%; *p* = 0.0013), the recommendation for children and adolescents to consume fish (84.6% vs. 69.8%; *p* < 0.0001), the recommendation to consume fish at least twice a week (39.0% vs. 30.4%; *p* = 0.0022), fish containing health-promoting fatty acids (84.0% vs. 73.2%; *p* < 0.0001), fish-derived fatty acids being good for health (75.1% vs. 61.8%; *p* < 0.0001), fish-derived fatty acids lowering blood cholesterol (45.9% vs. 38.8%; *p* = 0.0165), the risk of allergies connected to consuming fish (30.1% vs. 23.9%; *p* = 0.0201), the risk of bacteria and parasites in fish (57.5% vs. 44.5%; *p* < 0.0001), the risk of contaminants in fish (70.6% vs. 54.6%; *p* < 0.0001), and the risk of mercury in fish (42.5% vs. 26.3%; *p* < 0.0001).

Table 10 presents a graphical summary of fish-knowledge differences between the analyzed sub-groups in relation to the specific statements concerning fish-intake benefits and safety concerns.

## 4. Discussion

### 4.1. General Knowledge

In the present study, the recommendation for children and adolescents to consume fish was known to almost three-quarters of participants, while the recommendation to consume fish at least twice a week was known only to every third person (33.4%). Similarly, the answer concerning the recommendation for pregnant women to consume fish was known only to every third person and only to about 40% of the female respondents. Taking that into consideration there is a high risk that at the time when these adolescents become pregnant, they might still lack this knowledge and will not adhere to that recommendation. It corresponds with the fact that studies indicate that pregnant women often do not know the benefits of consuming fish, as well as that they frequently do not receive any advice to consume it [47]. On the contrary, they often receive advice from their physicians to limit fish intake [47]. Among pregnant women the awareness of fish-consumption risk associated with mercury is sometimes also more prevalent than of the benefits of omega-3 fatty acids found in fish [48]. Moreover, in Poland, fish intake among pregnant women is observed to be inadequate [49,50].

While not knowing that it is recommended for pregnant women to consume fish is not so surprising as the participants were adolescents, the fact that adolescents are not familiar with the basic general recommendation to consume fish at least two times per week is quite alarming. This lack of knowledge might have an implication for adolescents’ food choices as low fish and seafood intake in children and adolescents is commonly observed [51,52], and such low intake is more frequent among children and adolescents than in adults [53].

Based on the answers provided to the ‘dummy’ statement about fish being a source of fiber, it seems that many adolescents do not know that fish are not a source of fiber, as almost three-quarters of participants provided a wrong answer to that statement. However, the acquiescence bias should also be indicated here [54]. Participants might have confirmed because they could have made a wrong assumption that since fiber was good for health and so was fish, fish must be a good source of it. This nescience was also observed among adults—in Belgium 45.5% of the study participants believed that fish contain fiber [31]. On the other hand, two thirds of the respondents in the present study indicated that the fatty acids found in fish are good for health. This statement was a reverse one, so the risk of the acquiescence bias is, here, contradicted. In the Belgian study, the fact that omega-3 fatty acids have a positive influence on human health was known only to 30% of respondents [31], which is more than two times less than in the present study. It seems, therefore, that the misconception that fish contain fiber is much more common than the awareness of fish containing health-promoting fatty acids. However, in the present study, the knowledge on the type of fatty acids found in fish was very low; the majority of the adolescents identified them as ‘trans’ fatty acids, while more than every third declared having no opinion on it.

Regarding vitamin D, in the present study, independent of the characteristics of the participants, only every second person knew that fish are a source of it. Comparing that with the frequent misconception that fish contain fiber, it seems that many Polish adolescents do not associate fish with one of their main nutrients, namely omega-3 fatty acids and vitamin D, whose best food source for humans are fatty fish [55]. This is of great importance as it is known that vitamin D intake among Polish adolescents is very low [56,57]. Similarly, studies conducted in other countries show similar results concerning the knowledge that fish are a source of vitamin D—in Belgium this fact was indicated by 53.3% of the study participants [31].

Concerning the safety of fish consumption, the fact that eating fish may cause allergy was not widely known—only every fourth participant knew the correct answer regarding this statement. The possibility that fish may contain dioxins or mercury was not known by many either—only by every fifth and every third participant, respectively. In the Belgian study [31], the results were somehow similar—fewer people (29.1%) indicated dioxins as possible pollutants in fish compared to heavy metals (45.8%), one of which is mercury. However, in the present study more adolescents were aware of the fact that fish might contain bacteria and parasites or contaminants, as the proper answers were provided by almost half or more than half of respondents, respectively.

### 4.2. Determinants of Knowledge

According to the present study factors which were positively associated with the knowledge on fish health benefits and safety were female gender, being an adult, being underweight, living in an urban environment, living in a region far away from the sea and attending a comprehensive high school. Most of those variables are also indicated in other studies as variables which are associated with nutritional knowledge, including knowledge on fish. An Iranian study among primary and junior high-school pupils revealed very similar results, that the nutritional knowledge of females as well as junior high-school pupils and pupils from urban areas was higher than that of males, primary school children and pupils from rural areas [58]. In a study among adult Poles, it was age, as well as the education level, which was positively associated with objective knowledge on fish, which was assessed based on statements very similar to the present study (two false statements: ‘Fish is a source of dietary fiber’ and ‘Cod is a fatty fish’, and two true statements: ‘Fish is a source of omega-3 fatty acids’ and ‘Salmon is a fatty fish’) [35]. Moreover, in an English study analyzing nutritional knowledge among adults, it was women, people of higher education and occupational category, as well as people aged 35–64 who received higher knowledge scores than the other subgroups [59]. In a Canadian study, it was also women and participants with higher education whose nutritional-knowledge score was higher than in men or those with lower education [60]. This is in line with the present study concerning the female gender, but it also seems to be in accordance with adolescents attending comprehensive school and having better knowledge due to the fact that pupils who attend comprehensive schools in Poland choose higher education more frequently than pupils from vocational schools [61].

Similar to the present study, an Italian study among children and adolescents living in a rural area found that being older, as well as underweight or normal weight were linked to higher nutritional knowledge scores [62]. In the present study, the share of correct answers to some of the statements was significantly higher among underweight adolescents, followed by those of normal BMI, while the lowest share of correct answers in the present study was observed in the excessive body mass subgroup of participants. A study among adults in Cyprus also showed that the increase in nutritional knowledge is associated with having a lower BMI [63]. The results from the present study are also in line with a study among adolescents aged 17–19 from continental Croatia, which showed that boys, adolescents from rural areas, as well as overweight adolescents had lower nutritional knowledge than the other subgroups [64]. However, other studies are not always in line with these results and sometimes no association is stated or even a reverse association—a study among school-aged children from Ghana revealed that nutritional knowledge was weakly, but positively correlated with BMI for age [65].

Interestingly, the results of the present study are not in line with an Austrian study assessing nutritional knowledge among over five hundred secondary school adolescents regarding the place of residence [66]. In the cited study, pupils from rural regions scored better than the ones from urban regions, which is contrary to the results from the present study in which those from an urban environment provided correct answers more frequently. However, what should be noted is that in the cited study, the analysis was completed for pupils attending the so-called New Middle Schools in which a subject called ‘Nutrition and Household’ is taught (with varying frequency depending on the school), while in the present study, the analysis comprised students from the whole country, not only from one specific type of school and where no such dedicated subject is taught. In the Austrian study, more nutrition education classes were observed in rural areas which is probably the reason for better nutritional knowledge among students from these areas. Importantly, in the cited study, pupils who had more nutrition education classes in a week had higher nutritional-knowledge scores [66], which shows that nutrition education at school is an important factor linked to the nutritional knowledge of adolescents.

An important question is whether the higher knowledge on fish consumption among some subgroups observed in the present study, namely individuals of the female gender, adults, underweight participants, those living in an urban environment, participants living in regions far away from the sea and those attending a comprehensive high school, corresponds to a higher fish consumption among these subgroups. A Polish study found that among adolescents and young adults aged 15–29 there is a significant correlation between fish consumption and the degree of urbanization [67]. This result is in line with the result from the present study, in which adolescents from urban areas provided a higher share of correct answers to some of the analyzed statements than their counterparts from rural regions. A Russian cross-sectional study among adult participants also indicated that fish intake was statistically higher among participants from an urban area compared to a rural area [68]. Interestingly, the Russian study showed that the easy access to fish markets in the city might be a greater fish-consumption driver than a nearby body of water suitable for fishing, such as a lake [68].

However, higher nutritional knowledge concerning fish does not always seem to correspond to greater fish intake. Fish are usually eaten in larger quantities by men, not women, not in line with the results of the present study and the other studies which show that female adolescents’ knowledge regarding fish-intake benefits and safety risks is higher than male. According to the European Investigation into Cancer and Nutrition (EPIC) study, the intake of fish and fish products was higher among men than women almost in all analyzed countries and administrative centers [69]. A higher fish intake among men than among women was also observed in the Russian study, but it was statistically significant only for the rural area [68]. Interestingly, in the EPIC study, the crude mean fish and fish-products intake was higher for women than men only among the ‘health-conscious’ participants from the United Kingdom, and the adjusted for age, weighted for season and day of the week mean intake of fish and fish products was higher among women than men from the Ragusa region in Italy and again among the British health-conscious’ participants [69].

Concerning BMI, which according to the present study was associated negatively with the share of correct answers to some of the statements, it seems that fish intake is not correlated negatively with it. European longitudinal studies showed no relation between higher fish intake and lower body mass [70] or waist circumference [71], while a Japanese study showed a direct positive association between energy-adjusted fish intake and BMI [72]. However, it should be noted that the fish-intake patterns in Japan are very different from those in Europe [73], which could be the reason for the opposite results.

Moreover, according to the EPIC study [69], fish intake is usually higher in areas with greater access to the sea. This does not correspond to the results from the present study, in which adolescents from regions situated far away from the sea provided a higher share of correct answers to five of the twenty analyzed statements. However, what should also be noted is that the share of correct answers concerning the other fifteen statements was not different among those two sub-groups. Interestingly, while assessing the national data on fish intake in different regions of Poland, it turns out that the consumption in some of the regions situated far away from the sea may be even higher than in the seaside regions [74]. This difference in consumption but also the difference seen in knowledge in favor for the far-away-from-the-sea sub-group in the present study could, therefore, be linked to the fact that in Poland not only saltwater fish but also freshwater fish, especially carp, are consumed [75], and hence fish availability does not depend on the access to the sea only. What might also play a role is that the larger carp farms are distributed in the regions situated far away from the sea [75].

The fact that females as well as those who were underweight and those living in regions far away from sea provided a higher share of correct answers to some of the analyzed statements, hence their nutritional knowledge concerning fish seems to be higher, can be very beneficial to these subgroups but also to their relatives. Usually, it is women who are responsible for the household grocery shopping for the family [76]. Therefore, the better knowledge among females can, in the future, result in more frequent purchase and consumption of fish in their households [77]. Moreover, thanks to the more detailed knowledge, their choice of fish species and fish quality might also be better, and, therefore, it might result in a higher intake of high-quality nutritious fish among women, but also their spouses and children. Similar assumptions apply to those whose BMI indicated they are underweight—by dint of their more extensive knowledge concerning fish-intake benefits and safety concerns, their motivation to consume fish might also be higher, hence a lower risk of malnutrition. On the other hand, adolescents from rural areas as well as those who attend vocational schools might be especially at risk of not consuming fish or consuming fish of poor quality because of their insufficient knowledge concerning fish-intake benefits and safety risks. This is especially alarming since studies show that adolescents from rural areas have a greater risk of obesity and physical inactivity [78,79], as well as that their diet is often of poor quality as they commonly prefer fast food to fruit and vegetables, frequently consume sweets and consume few meals during the day [80].

## 5. Conclusions

In the studied population-based sample of Polish adolescents, their knowledge concerning fish-consumption benefits and safety was in many cases inadequate. Males, minor adolescents, normal weight and excessive body mass adolescents, adolescents from rural regions and from regions by the sea, as well as those attending vocational schools provided a lower share of correct answers to some of the analyzed statements, hence nutrition education concerning fish-consumption benefits and safety should be targeted primarily at these subgroups of adolescents. Importantly, the recommendation for children and adolescents to consume fish at least two times per week should also be addressed, since according to the present study it is not known by all.

## Figures and Tables

**Table 1:** Statements concerning knowledge about fish used in the present questionnaire and the correct answers.

No.	Statement	Correct Answer
1	Fish are a good source of protein.	True
2	Fish contain a lot of fiber.	False
3	Fish are a good source of vitamin D.	True
4	Fish contain a lot of unhealthy fats.	False
5	Fish have good fat.	True
6	Eating fish is good for the heart.	True
7	Eating fish is not good for the brain.	False
8	Eating fish is good for you.	True
9	Fish contain a lot of healthy ‘trans’ fats.	False
10	Eating fish lowers cholesterol.	True
11	Fish are a good source of vitamin C.	False
12	Eating fish may cause allergies.	True
13	Fish may contain bacteria or parasites.	True
14	Children and adolescents should not eat fish.	False
15	Eating fish is recommended for pregnant women.	True
16	Fish may contain contaminants.	True
17	Fish should be eaten once a week at the most.	False
18	Fish may contain polychlorinated biphenyls (PCBs).	True
19	Cod is a fatty fish.	False
20	Fish may contain mercury.	True

**Table 2:** Characteristics of the studied group.

	All		Females		Males	
Variable	Mean ± SD	Median (Min–Max)	Mean ± SD	Median (Min–Max)	Mean ± SD	Median (Min–Max)
Age, years	16.7 ± 1.2	17 (14–22)	16.8 ± 1.2	17 (14–22)	16.5 ± 1.3	16 (14–21)
Height, cm	170.3 ± 8.9	170.0 (150.0–200.0)	166.0 ± 6.2	166.0 (150.0–185.0)	178.7 ± 7.3	179.0 (150.0–200.0)
Weight, kg	63.7 ± 13.8	60.0 (35.0–120.0)	59.1 ± 10.9	57.0 (35.0–115.0)	72.6 ± 14.5	70.0 (40.0–120.0)

**Table 3:** The number of correct and incorrect answers provided by the population-based sample of Polish secondary school students within the studied issues of fish-consumption benefits and safety concerns.

Studied Knowledge	Studied Statement	Answers Provided by Studied Adolescents (*n* = 1289)	
		Correct	Incorrect
Content of nutrients in fish	Source of protein (statement 1)	1017 (78.9%)	272 (21.1%)
	Not a source of fiber (statement 2)	386 (29.9%)	903 (70.1%)
	Some fish have more fat than others (statement 19)	287 (22.3%)	1002 (77.7%)
	Not a source of ‘trans’ fatty acids (statement 9)	98 (7.6%)	1191 (92.4%)
	Source of vitamin D (statement 3)	714 (55.4%)	575 (44.6%)
	Not a source of vitamin C (statement 11)	383 (29.7%)	906 (70.3%)
General health influence of fish consumption	Good for health (statement 8)	917 (71.1%)	372 (28.9%)
	Good for the heart (statement 6)	829 (64.3%)	460 (35.7%)
	Good for the brain (statement 7)	845 (65.6%)	444 (34.4%)
	Recommended to be consumed by children/adolescents (statement 14)	966 (74.9%)	323 (25.1%)
	Recommended to be consumed by pregnant women (statement 15)	463 (35.9%)	826 (64.1%)
	Recommended to be consumed at least twice a week (statement 17)	430 (33.4%)	859 (66.6%)
Health influence of fish-derived fats	Health-promoting fatty acids (statement 5)	992 (77.0%)	297 (23.0%)
	Fish-derived fatty acids good for health (statement 4)	856 (66.4%)	433 (33.6%)
	Fish-derived fatty acids lower blood cholesterol (statement 10)	532 (41.3%)	757 (58.7%)
Safety concerns	Risk of allergies (statement 12)	336 (26.1%)	953 (73.9%)
	Risk of bacteria and parasites in fish (statement 13)	632 (49.0%)	657 (51.0%)
	Risk of contaminants in fish (statement 16)	776 (60.2%)	513 (39.8%)
	Risk of polychlorinated biphenyls (PCBs) in fish (statement 18)	264 (20.5%)	1025 (79.5%)
	Risk of mercury in fish (statement 20)	412 (32.0%)	877 (68.0%)

**Table 4:** The number of correct and incorrect answers provided by the population-based sample of Polish secondary school students within the studied issues of fish-consumption benefits and safety concerns in sub-groups stratified by gender.

Studied Knowledge	Studied Statement	Answers Provided by Studied Adolescents (*n* = 1289)				*p*
		Female (*n* = 849)		Male (*n* = 440)		
		Correct	Incorrect	Correct	Incorrect	
Content of nutrients in fish	Source of protein (statement 1)	690 (81.3%)	159 (18.7%)	327 (74.3%)	113 (25.7%)	0.0047
	Not a source of fiber (statement 2)	256 (30.2%)	593 (69.8%)	130 (29.5%)	310 (70.5%)	0.8715
	Some fish have more fat than others (statement 19)	196 (23.1%)	653 (76.9%)	91 (20.7%)	349 (79.3%)	0.3611
	Not a source of ‘trans’ fatty acids (statement 9)	69 (8.1%)	780 (91.9%)	29 (6.6%)	411 (93.4%)	0.3811
	Source of vitamin D (statement 3)	471 (55.5%)	378 (44.5%)	243 (55.2%)	197 (44.8%)	0.9789
	Not a source of vitamin C (statement 11)	265 (31.2%)	584 (68.8%)	118 (26.8%)	322 (73.2%)	0.1157
General health influence of fish consumption	Good for health (statement 8)	627 (73.9%)	222 (26.1%)	290 (65.9%)	150 (34.1%)	0.0035
	Good for the heart (statement 6)	563 (66.3%)	286 (33.7%)	266 (60.5%)	174 (39.5%)	0.0433
	Good for the brain (statement 7)	562 (66.2%)	287 (33.8%)	283 (64.3%)	157 (35.7%)	0.5414
	Recommended to be consumed by children/adolescents (statement 14)	673 (79.3%)	176 (20.7%)	293 (66.6%)	147 (33.4%)	<0.0001
	Recommended to be consumed by pregnant women (statement 15)	335 (39.5%)	514 (60.5%)	128 (29.1%)	312 (70.9%)	0.0003
	Recommended to be consumed at least twice a week (statement 17)	273 (32.2%)	576 (67.8%)	157 (35.7%)	283 (64.3%)	0.2259
Health influence of fish-derived fats	Health-promoting fatty acids (statement 5)	667 (78.6%)	182 (21.4%)	325 (73.9%)	115 (26.1%)	0.0672
	Fish-derived fatty acids good for health (statement 4)	571 (67.3%)	278 (32.7%)	285 (64.8%)	155 (35.2%)	0.4050
	Fish-derived fatty acids lower blood cholesterol (statement 10)	351 (41.3%)	498 (58.7%)	181 (41.1%)	259 (58.9%)	0.9907
Safety concerns	Risk of allergies (statement 12)	230 (27.1%)	619 (72.9%)	106 (24.1%)	334 (75.9%)	0.2729
	Risk of bacteria and parasites in fish (statement 13)	427 (50.3%)	422 (49.7%)	205 (46.6%)	235 (53.4%)	0.2292
	Risk of contaminants in fish (statement 16)	513 (60.4%)	336 (39.6%)	263 (59.8%)	177 (40.2%)	0.8678
	Risk of polychlorinated biphenyls (PCBs) in fish (statement 18)	155 (18.3%)	694 (81.7%)	109 (24.8%)	331 (75.2%)	0.0075
	Risk of mercury in fish (statement 20)	286 (33.7%)	563 (66.3%)	126 (28.6%)	314 (71.4%)	0.0750

**Table 5:** The number of correct and incorrect answers provided by the population-based sample of Polish secondary school students within the studied issues of fish-consumption benefits and safety concerns in sub-groups stratified by age.

Studied Knowledge	Studied Statement	Answers Provided by Studied Adolescents (*n* = 1289)				*p*
		Minors (*n* = 992)		Adults (*n* = 297)		
		Correct	Incorrect	Correct	Incorrect	
Content of nutrients in fish	Source of protein (statement 1)	766 (77.2%)	226 (22.8%)	251 (84.5%)	46 (15.5%)	0.0088
	Not a source of fiber (statement 2)	289 (29.1%)	703 (70.9%)	97 (32.7%)	200 (67.3%)	0.2749
	Some fish have more fat than others (statement 19)	208 (21.0%)	784 (79.0%)	79 (26.6%)	218 (73.4%)	0.0492
	Not a source of ‘trans’ fatty acids (statement 9)	70 (7.1%)	922 (92.9%)	28 (9.4%)	269 (90.6%)	0.2195
	Source of vitamin D (statement 3)	537 (54.1%)	455 (45.9%)	177 (59.6%)	120 (40.4%)	0.1107
	Not a source of vitamin C (statement 11)	283 (28.5%)	709 (71.5%)	100 (33.7%)	197 (66.3%)	0.1034
General health influence of fish consumption	Good for health (statement 8)	683 (68.9%)	309 (31.1%)	234 (78.8%)	63 (21.2%)	0.0012
	Good for the heart (statement 6)	619 (62.4%)	373 (37.6%)	210 (70.7%)	87 (29.3%)	0.0107
	Good for the brain (statement 7)	635 (64.0%)	357 (36.0%)	210 (70.7%)	87 (29.3%)	0.0394
	Recommended to be consumed by children/adolescents (statement 14)	733 (73.9%)	259 (26.1%)	233 (78.5%)	64 (21.5%)	0.1299
	Recommended to be consumed by pregnant women (statement 15)	345 (34.8%)	647 (65.2%)	118 (39.7%)	179 (60.3%)	0.1358
	Recommended to be consumed at least twice a week (statement 17)	311 (31.4%)	681 (68.6%)	119 (40.1%)	178 (59.9%)	0.0064
Health influence of fish-derived fats	Health-promoting fatty acids (statement 5)	753 (75.9%)	239 (24.1%)	239 (80.5%)	58 (19.5%)	0.1187
	Fish-derived fatty acids good for health (statement 4)	647 (65.2%)	345 (34.8%)	209 (70.4%)	88 (29.6%)	0.1146
	Fish-derived fatty acids lower blood cholesterol (statement 10)	396 (39.9%)	596 (60.1%)	136 (45.8%)	161 (54.2%)	0.0826
Safety concerns	Risk of allergies (statement 12)	270 (27.2%)	722 (72.8%)	66 (22.2%)	231 (77.8%)	0.1000
	Risk of bacteria and parasites in fish (statement 13)	491 (49.5%)	501 (50.5%)	141 (47.5%)	156 (52.5%)	0.5857
	Risk of contaminants in fish (statement 16)	587 (59.2%)	405 (40.8%)	189 (63.6%)	108 (36.4%)	0.1899
	Risk of polychlorinated biphenyls (PCBs) in fish (statement 18)	195 (19.7%)	797 (80.3%)	69 (23.2%)	228 (76.8%)	0.2086
	Risk of mercury in fish (statement 20)	316 (31.9%)	676 (68.1%)	96 (32.3%)	201 (67.7%)	0.9355

**Table 6:** The number of correct and incorrect answers provided by the population-based sample of Polish secondary school students within the studied issues of fish-consumption benefits and safety concerns in sub-groups stratified by the body mass of the studied adolescents.

Studied Knowledge	Studied Statement	Answers Provided by Studied Adolescents (*n* = 1289)						*p*
		Underweight (*n* = 90)		Proper Body Mass (*n* = 899)		Excessive Body Mass (*n* = 300)		
		Correct	Incorrect	Correct	Incorrect	Correct	Incorrect	
Content of nutrients in fish	Source of protein (statement 1)	77 (85.6%)	13 (14.4%)	695 (77.3%)	204 (22.7%)	245 (81.7%)	55 (18.3%)	0.0765
	Not a source of fiber (statement 2)	30 (33.3%)	60 (66.7%)	278 (30.9%)	621 (69.1%)	78 (26.0%)	222 (74.0%)	0.2093
	Some fish have more fat than others (statement 19)	25 (27.8%)	65 (72.2%)	210 (23.4%)	689 (76.6%)	52 (17.3%)	248 (82.7%)	0.0404
	Not a source of ‘trans’ fatty acids (statement 9)	8 (8.9%)	82 (91.1%)	75 (8.3%)	824 (91.7%)	15 (5.0%)	285 (95.0%)	0.1492
	Source of vitamin D (statement 3)	51 (56.7%)	39 (43.3%)	492 (54.7%)	407 (45.3%)	171 (57.0%)	129 (43.0%)	0.7658
	Not a source of vitamin C (statement 11)	32 (35.6%)	58 (64.4%)	286 (31.8%)	613 (68.2%)	65 (21.7%)	235 (78.3%)	0.0018
General health influence of fish consumption	Good for health (statement 8)	63 (70.0%)	27 (30.0%)	644 (71.6%)	255 (28.4%)	210 (70.0%)	90 (30.0%)	0.8377
	Good for the heart (statement 6)	58 (64.4%)	32 (35.6%)	588 (65.4%)	311 (34.6%)	183 (61.0%)	117 (39.0%)	0.3861
	Good for the brain (statement 7)	54 (60.0%)	36 (40.0%)	606 (67.4%)	293 (32.6%)	185 (61.7%)	115 (38.3%)	0.1000
	Recommended to be consumed by children/adolescents (statement 14)	68 (75.6%)	22 (24.4%)	682 (75.9%)	217 (24.1%)	216 (72.0%)	84 (28.0%)	0.4053
	Recommended to be consumed by pregnant women (statement 15)	31 (34.4%)	59 (65.6%)	318 (35.4%)	581 (64.6%)	114 (38.0%)	186 (62.0%)	0.6818
	Recommended to be consumed at least twice a week (statement 17)	25 (27.8%)	65 (72.2%)	313 (34.8%)	586 (65.2%)	92 (30.7%)	208 (69.3%)	0.2124
Health influence of fish-derived fats	Health-promoting fatty acids (statement 5)	66 (73.3%)	24 (26.7%)	701 (78.0%)	198 (22.0%)	225 (75.0%)	75 (25.0%)	0.3984
	Fish-derived fatty acids good for health (statement 4)	59 (65.6%)	31 (34.4%)	603 (67.1%)	296 (32.9%)	194 (64.7%)	106 (35.3%)	0.7349
	Fish-derived fatty acids lower blood cholesterol (statement 10)	36 (40.0%)	54 (60.0%)	373 (41.5%)	526 (58.5%)	123 (41.0%)	177 (59.0%)	0.9575
Safety concerns	Risk of allergies (statement 12)	20 (22.2%)	70 (77.8%)	234 (26.0%)	665 (74.0%)	82 (27.3%)	218 (72.7%)	0.6248
	Risk of bacteria and parasites in fish (statement 13)	41 (45.6%)	49 (54.4%)	446 (49.6%)	453 (50.4%)	145 (48.3%)	155 (51.7%)	0.7355
	Risk of contaminants in fish (statement 16)	49 (54.4%)	41 (45.6%)	558 (62.1%)	341 (37.9%)	169 (56.3%)	131 (43.7%)	0.1093
	Risk of polychlorinated biphenyls (PCBs) in fish (statement 18)	19 (21.1%)	71 (78.9%)	188 (20.9%)	711 (79.1%)	57 (19.0%)	243 (81.0%)	0.7678
	Risk of mercury in fish (statement 20)	29 (32.2%)	61 (67.8%)	293 (32.6%)	606 (67.4%)	90 (30.0%)	210 (70.0%)	0.7055

**Table 7:** The number of correct and incorrect answers provided by the population-based sample of Polish secondary school students within the studied issues of fish-consumption benefits and safety concerns in sub-groups stratified by rural/urban environment.

Studied Knowledge	Studied Statement	Answers Provided by Studied Adolescents (*n* = 1289)				*p*
		Rural Environment (*n* = 678)		Urban Environment (*n* = 611)		
		Correct	Incorrect	Correct	Incorrect	
Content of nutrients in fish	Source of protein (statement 1)	540 (79.6%)	138 (20.4%)	477 (78.1%)	134 (21.9%)	0.5322
	Not a source of fiber (statement 2)	196 (28.9%)	482 (71.1%)	190 (31.1%)	421 (68.9%)	0.4263
	Some fish have more fat than others (statement 19)	134 (19.8%)	544 (80.2%)	153 (25.0%)	458 (75.0%)	0.0273
	Not a source of ‘trans’ fatty acids (statement 9)	49 (7.2%)	629 (92.8%)	49 (8.0%)	562 (92.0%)	0.6667
	Source of vitamin D (statement 3)	367 (54.1%)	311 (45.9%)	347 (56.8%)	264 (43.2%)	0.3660
	Not a source of vitamin C (statement 11)	187 (27.6%)	491 (72.4%)	196 (32.1%)	415 (67.9%)	0.0885
General health influence of fish consumption	Good for health (statement 8)	472 (69.6%)	206 (30.4%)	445 (72.8%)	166 (27.2%)	0.2261
	Good for the heart (statement 6)	423 (62.4%)	255 (37.6%)	406 (66.4%)	205 (33.6%)	0.1441
	Good for the brain (statement 7)	417 (61.5%)	261 (38.5%)	428 (70.0%)	183 (30.0%)	0.0016
	Recommended to be consumed by children/adolescents (statement 14)	495 (73.0%)	183 (27.0%)	471 (77.1%)	140 (22.9%)	0.1047
	Recommended to be consumed by pregnant women (statement 15)	236 (34.8%)	442 (65.2%)	227 (37.2%)	384 (62.8%)	0.4135
	Recommended to be consumed at least twice a week (statement 17)	218 (32.2%)	460 (67.8%)	212 (34.7%)	399 (65.3%)	0.3638
Health influence of fish-derived fats	Health-promoting fatty acids (statement 5)	507 (74.8%)	171 (25.2%)	485 (79.4%)	126 (20.6%)	0.0585
	Fish-derived fatty acids good for health (statement 4)	427 (63.0%)	251 (37.0%)	429 (70.2%)	182 (29.8%)	0.0072
	Fish-derived fatty acids lower blood cholesterol (statement 10)	276 (40.7%)	402 (59.3%)	256 (41.9%)	355 (58.1%)	0.7063
Safety concerns	Risk of allergies (statement 12)	173 (25.5%)	505 (74.5%)	163 (26.7%)	448 (73.3%)	0.6813
	Risk of bacteria and parasites in fish (statement 13)	321 (47.3%)	357 (52.7%)	311 (50.9%)	300 (49.1%)	0.2228
	Risk of contaminants in fish (statement 16)	380 (56.0%)	298 (44.0%)	396 (64.8%)	215 (35.2%)	0.0016
	Risk of polychlorinated biphenyls (PCBs) in fish (statement 18)	133 (19.6%)	545 (80.4%)	131 (21.4%)	480 (78.6%)	0.4587
	Risk of mercury in fish (statement 20)	206 (30.4%)	472 (69.6%)	206 (33.7%)	405 (66.3%)	0.2221

**Table 8:** The number of correct and incorrect answers provided by the population-based sample of Polish secondary school students within the studied issues of fish-consumption benefits and safety concerns in sub-groups stratified by the location of the region of residence in relation to the Baltic Sea.

Studied Knowledge	Studied Statement	Answers Provided by Studied Adolescents (*n* = 1289)				*p*
		Region Situated by the Sea (*n* = 384)		Region Situated Far away from the Sea (*n* = 905)		
		Correct	Incorrect	Correct	Incorrect	
Content of nutrients in fish	Source of protein (statement 1)	310 (80.7%)	74 (19.3%)	707 (78.1%)	198 (21.9%)	0.3297
	Not a source of fiber (statement 2)	85 (22.1%)	299 (77.9%)	301 (33.3%)	604 (66.7%)	0.0001
	Some fish have more fat than others (statement 19)	62 (16.1%)	322 (83.9%)	225 (24.9%)	680 (75.1%)	0.0008
	Not a source of ‘trans’ fatty acids (statement 9)	29 (7.6%)	355 (92.4%)	69 (7.6%)	836 (92.4%)	1.0000
	Source of vitamin D (statement 3)	218 (56.8%)	166 (43.2%)	496 (54.8%)	409 (45.2%)	0.5568
	Not a source of vitamin C (statement 11)	85 (22.1%)	299 (77.9%)	298 (32.9%)	607 (67.1%)	0.0001
General health influence of fish consumption	Good for health (statement 8)	270 (70.3%)	114 (29.7%)	647 (71.5%)	258 (28.5%)	0.7188
	Good for the heart (statement 6)	247 (64.3%)	137 (35.7%)	582 (64.3%)	323 (35.7%)	1.0000
	Good for the brain (statement 7)	236 (61.5%)	148 (38.5%)	609 (67.3%)	296 (32.7%)	0.0509
	Recommended to be consumed by children/adolescents (statement 14)	264 (68.8%)	120 (31.3%)	702 (77.6%)	203 (22.4%)	0.0011
	Recommended to be consumed by pregnant women (statement 15)	141 (36.7%)	243 (63.3%)	322 (35.6%)	583 (64.4%)	0.7443
	Recommended to be consumed at least twice a week (statement 17)	118 (30.7%)	266 (69.3%)	312 (34.5%)	593 (65.5%)	0.2150
Health influence of fish-derived fats	Health-promoting fatty acids (statement 5)	287 (74.7%)	97 (25.3%)	705 (77.9%)	200 (22.1%)	0.2460
	Fish-derived fatty acids good for health (statement 4)	243 (63.3%)	141 (36.7%)	613 (67.7%)	292 (32.3%)	0.1379
	Fish-derived fatty acids lower blood cholesterol (statement 10)	148 (38.5%)	236 (61.5%)	384 (42.4%)	521 (57.6%)	0.2167
Safety concerns	Risk of allergies (statement 12)	96 (25.0%)	288 (75.0%)	240 (26.5%)	665 (73.5%)	0.6178
	Risk of bacteria and parasites in fish (statement 13)	179 (46.6%)	205 (53.4%)	453 (50.1%)	452 (49.9%)	0.2850
	Risk of contaminants in fish (statement 16)	224 (58.3%)	160 (41.7%)	552 (61.0%)	353 (39.0%)	0.4063
	Risk of polychlorinated biphenyls (PCBs) in fish (statement 18)	70 (18.2%)	314 (81.8%)	194 (21.4%)	711 (78.6%)	0.2189
	Risk of mercury in fish (statement 20)	92 (24.0%)	292 (76.0%)	320 (35.4%)	585 (64.6%)	0.0001

**Table 9:** The number of correct and incorrect answers provided by the population-based sample of Polish secondary school students within the studied issues of fish-consumption benefits and safety concerns in sub-groups stratified by the type of school.

Studied Knowledge	Studied Statement	Answers Provided by Studied Adolescents (*n* = 1289)				*p*
		Comprehensive School (*n* = 449)		Vocational School (*n* = 840)		
		Correct	Incorrect	Correct	Incorrect	
Content of nutrients in fish	Source of protein (statement 1)	362 (80.6%)	87 (19.4%)	655 (78.0%)	185 (22.0%)	0.2992
	Not a source of fiber (statement 2)	170 (37.9%)	279 (62.1%)	216 (25.7%)	624 (74.3%)	<0.0001
	Some fish have more fat than others (statement 19)	152 (33.9%)	297 (66.1%)	135 (16.1%)	705 (83.9%)	<0.0001
	Not a source of ‘trans’ fatty acids (statement 9)	34 (7.6%)	415 (92.4%)	64 (7.6%)	776 (92.4%)	1.0000
	Source of vitamin D (statement 3)	265 (59.0%)	184 (41.0%)	449 (53.5%)	391 (46.5%)	0.0633
	Not a source of vitamin C (statement 11)	171 (38.1%)	278 (61.9%)	212 (25.2%)	628 (74.8%)	<0.0001
General health influence of fish consumption	Good for health (statement 8)	330 (73.5%)	119 (26.5%)	587 (69.9%)	253 (30.1%)	0.1934
	Good for the heart (statement 6)	307 (68.4%)	142 (31.6%)	522 (62.1%)	318 (37.9%)	0.0305
	Good for the brain (statement 7)	321 (71.5%)	128 (28.5%)	524 (62.4%)	316 (37.6%)	0.0013
	Recommended to be consumed by children/adolescents (statement 14)	380 (84.6%)	69 (15.4%)	586 (69.8%)	254 (30.2%)	<0.0001
	Recommended to be consumed by pregnant women (statement 15)	164 (36.5%)	285 (63.5%)	299 (35.6%)	541 (64.4%)	0.7866
	Recommended to be consumed at least twice a week (statement 17)	175 (39.0%)	274 (61.0%)	255 (30.4%)	585 (69.6%)	0.0022
Health influence of fish-derived fats	Health-promoting fatty acids (statement 5)	377 (84.0%)	72 (16.0%)	615 (73.2%)	225 (26.8%)	<0.0001
	Fish-derived fatty acids good for health (statement 4)	337 (75.1%)	112 (24.9%)	519 (61.8%)	321 (38.2%)	<0.0001
	Fish-derived fatty acids lower blood cholesterol (statement 10)	206 (45.9%)	243 (54.1%)	326 (38.8%)	514 (61.2%)	0.0165
Safety concerns	Risk of allergies (statement 12)	135 (30.1%)	314 (69.9%)	201 (23.9%)	639 (76.1%)	0.0201
	Risk of bacteria and parasites in fish (statement 13)	258 (57.5%)	191 (42.5%)	374 (44.5%)	466 (55.5%)	<0.0001
	Risk of contaminants in fish (statement 16)	317 (70.6%)	132 (29.4%)	459 (54.6%)	381 (45.4%)	<0.0001
	Risk of polychlorinated biphenyls (PCBs) in fish (statement 18)	104 (23.2%)	345 (76.8%)	160 (19.0%)	680 (81.0%)	0.0946
	Risk of mercury in fish (statement 20)	191 (42.5%)	258 (57.5%)	221 (26.3%)	619 (73.7%)	<0.0001

**Table 10:** Graphical summary of fish knowledge differences between the analyzed sub-groups in relation to the specific statements concerning fish-intake benefits and safety concerns.

Studied Knowledge	Studied Statement	Gender	Age	Body Mass	Environment	Region	Type of School
Content of nutrients in fish	Source of protein (statement 1)	Female > Male	Minors < Adults				
	Not a source of fiber (statement 2)					By the sea < Far away from	Comprehensive > Vocational school
	Some fish have more fat than others (statement 19)		Minors < Adults	Underweight > Proper body mass > Excessive body mass	Rural < Urban	By the sea < Far away from	Comprehensive > Vocational school
	Not a source of ‘trans’ fatty acids (statement 9)						
	Source of vitamin D (statement 3)						
	Not a source of vitamin C (statement 11)			Underweight > Proper body mass > Excessive body mass		By the sea < Far away from	Comprehensive > Vocational school
General health influence of fish consumption	Good for health (statement 8)	Female > Male	Minors < Adults				
	Good for the heart (statement 6)	Female > Male	Minors < Adults				Comprehensive > Vocational school
	Good for the brain (statement 7)		Minors < Adults		Rural < Urban		Comprehensive > Vocational school
	Recommended to be consumed by children/adolescents (statement 14)	Female > Male				By the sea < Far away from	Comprehensive > Vocational school
	Recommended to be consumed by pregnant women (statement 15)	Female > Male					
	Recommended to be consumed at least twice a week (statement 17)		Minors < Adults				Comprehensive > Vocational school
Health influence of fish-derived fats	Health-promoting fatty acids (statement 5)						Comprehensive > Vocational school
	Fish-derived fatty acids good for health (statement 4)				Rural < Urban		Comprehensive > Vocational school
	Fish-derived fatty acids lower blood cholesterol (statement 10)						Comprehensive > Vocational school
Safety concerns	Risk of allergies (statement 12)						Comprehensive > Vocational school
	Risk of bacteria and parasites in fish (statement 13)						Comprehensive > Vocational school
	Risk of contaminants in fish (statement 16)				Rural < Urban		Comprehensive > Vocational school
	Risk of polychlorinated biphenyls (PCBs) in fish (statement 18)	Female < Male					
	Risk of mercury in fish (statement 20)					By the sea < Far away from	Comprehensive > Vocational school

## Data Availability

Data available on request.
